# Chebulic Acid Prevents Methylglyoxal-Induced Mitochondrial Dysfunction in INS-1 Pancreatic β-Cells

**DOI:** 10.3390/antiox9090771

**Published:** 2020-08-20

**Authors:** Hyun-jung Yoo, Chung-Oui Hong, Sang Keun Ha, Kwang-Won Lee

**Affiliations:** 1 Department of Biotechnology, College of Life Science & Biotechnology, Korea University, Seoul 02841, Korea; maryyu031@korea.ac.kr (H.-j.Y.); hco1207@korea.kr (C.-O.H.); 2 Research Division of Food Functionality, Korea Food Research Institute, Wanju-gun, Jeollabuk-do 55365, Korea; skha@kfri.re.kr

**Keywords:** chebulic acid, methylglyoxal, INS-1 cells, mitochondrial dysfunction, insulin secretion

## Abstract

To investigate the anti-diabetic properties of chebulic acid (CA) associated with the prevention of methyl glyoxal (MG)-induced mitochondrial dysfunction in INS-1 pancreatic β-cells, INS-1 cells were pre-treated with CA (0.5, 1.0, and 2.0 μM) for 48 h and then treated with 2 mM MG for 8 h. The effects of CA and MG on INS-1 cells were evaluated using the following: 3-(4,5-dimethylthiazol-2-yl)-2,5-diphenyltetrazolium bromide (MTT) assay; glyoxalase 1 (Glo-1) expression via Western blot and enzyme activity assays; Nrf-2, nuclear factor erythroid 2-related factor 2 protein expression via Western blot assay; reactive oxygen species (ROS) production assay; mRNA expression of mitochondrial dysfunction related components (UCP2, uncoupling protein 2; VDAC1, voltage-dependent anion-selective channel-1; cyt *c*, cytochrome *c* via quantitative reverse transcriptase-PCR; mitochondrial membrane potential (MMP); adenosine triphosphate (ATP) synthesis; glucose-stimulated insulin secretion (GSIS) assay. The viability of INS-1 cells was maintained upon pre-treating with CA before exposure to MG. CA upregulated Glo-1 protein expression and enzyme activity in INS-1 cells and prevented MG-induced ROS production. Mitochondrial dysfunction was alleviated by CA pretreatment; this occurred via the downregulation of UCP2, VDAC1, and cyt *c* mRNA expression and the increase of MMP and ATP synthesis. Further, CA pre-treatment promoted the recovery from MG-induced decrease in GSIS. These results indicated that CA could be employed as a therapeutic agent in diabetes due to its ability to prevent MG-induced development of insulin sensitivity and oxidative stress-induced dysfunction of β-cells.

## 1. Introduction

Diabetes mellitus is a serious chronic metabolic illness with increasing global incidence that presents a major public health concern. According to the 2016 survey of the WHO, 8.5% of the adult population suffers from diabetes, and this incidence is rapidly increasing [1]. Ninety percent of the patients with diabetes suffer from type 2 diabetes, and one of the main causes of this disease is reduced insulin secretion due to pancreatic β-cell dysfunction [2].

Methylglyoxal (MG) is a reactive dicarbonyl compound formed during glycolysis. It is produced during conditions of dicarbonyl stress and is a precursor of advanced glycation end products (AGEs), which trigger aging [3]. Some studies have shown that MG concentrations are highly increased in patients with diabetes and that increased MG levels can damage the tissues and cells [4]. The glyoxalase system is a MG detoxification system that is present in the cytosol of all mammalian cells [5]. It is comprised of glyoxalase-1 (Glo-1), Glo-2, and reduced glutathione (GSH). Glo-1 catalyzes the conversion of hemithioacetal adduct of MG with GSH into the intermediate S-d-lactoylglutathione (SLG), and finally Glo-2 degrades it to lactate [6]. Rabbani et al. [6] reported that dicarbonyl stress is a pathological factor associated with obesity, diabetes complications, and aging, and that it may be alleviated by increasing Glo-1 expression and dicarbonyl scavenger levels.

Mitochondrial dysfunction is a major disorder induced by oxidative stress and is commonly observed in diabetes. Excessive oxidative stress in β-cells causes disorders in mitochondrial membrane components including the respiratory chain via proton leaks and inhibition of adenosine triphosphate (ATP) synthesis, ultimately resulting in reduced insulin secretion. Pancreatic islets are more susceptible than other tissues to these changes because of the low contents of antioxidants like superoxide dismutase (SOD) and GSH [7,8]. As exposure to MG results in the production of many reactive oxygen species (ROS), antioxidant therapy is required as part of diabetes management [9].

**Figure 1:**
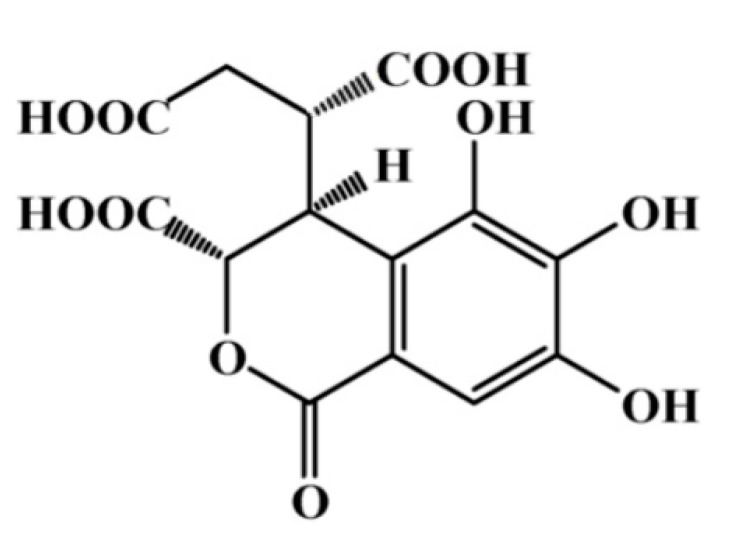
Chemical structure of chebulic acid (CA), according to Lee et al. [10].

Our previous studies have demonstrated the effects of chebulic acid (CA, Figure 1), a phenolic compound isolated from the fruit of *Terminalia chebula* Retz, which exhibits anti-oxidant, anti-fibrotic, anti-inflammatory, and anti-glycative effects [10,11,12]. Recently, we verified the effect of CA on alveolar epithelial damage arising from exposure to urban particle matter [13]. However, the preventive effect of CA with respect to pancreatic β-cell damage remains unknown. Therefore, the present study investigated whether CA can prevent MG-induced mitochondrial dysfunction and impaired insulin secretion by the INS-1 pancreatic β-cells.

## 2. Materials and Methods

### 2.1. Chemicals

RPMI-1640 medium and RIPA buffer were purchased from ThermoFisher Inc. (Carlsbad, CA, USA). Fetal bovine serum (FBS), trypsin, and penicillin-streptomycin were purchased from HyClone (Logan, UT, USA). The primary antibodies were anti-glyceraldehyde-3-phosphate dehydrogenase (GAPDH) monoclonal antibody (sc-32233; Santa Cruz Biotechnology, Santa Cruz, CA, USA), anti-Glo-1 monoclonal antibody (sc-133214; Santa Cruz Biotechnology, Santa Cruz, CA, USA), and anti-nuclear factor erythroid 2-related factor 2 (Nrf-2) monoclonal antibody (sc-722; Santa Cruz Biotechnology, Santa Cruz, CA, USA). The secondary antibodies were mouse anti-rabbit IgG-HRP (sc-2357; Santa Cruz Biotechnology, Santa Cruz, CA, USA) and goat anti-mouse IgG-HRP (AP124P; Sigma-Aldrich, St. Louis, MO, USA). JC-1, 5,5′,6,6′-tetrachloro-1,1′,3,3′-tetraethylbenzimidazolylcarbocyanine chloride was purchased from Biotium (Fremont, CA, USA). MG, N-acetyl-cysteine (NAC), and all other chemicals were purchased from Sigma-Aldrich.

### 2.2. Preparation of Chebulic Acid (CA)

CA was isolated from the fruit of dried *Terminalia chebula* purchased from a market (Kyung-dong Herb-Market, Seoul, Korea). The procedures by which CA was isolated from *Terminalia chebula* have been described previously by Lee et al. [10].

### 2.3. Cell Culture

Rat INS-1 pancreatic β-cells (INS-1) were obtained from AddexBio Technologies (San Diego, CA, USA) and cultured in RPMI-1640 medium (pH 7.2) containing 10% FBS, 1.5 g/L NaHCO_3_, 10 mM 4-(2-hydroxyethyl)-1-piperazineethane sulfonic acid (HEPES), 0.11 g/L sodium pyruvate, 50 μM 2-mercaptoethanol, and 100 units/mL of penicillin and streptomycin. Cells were maintained in an incubator containing 5% CO_2_ at 37 °C. For the following experiments, cells were pre-treated with CA (0.5, 1.0, and 2.0 μM) dissolved in DMSO, 0.5 mM NAC, and the culture medium for 48 h. After pre-treatment, cells were washed once with phosphate buffered saline (PBS) and then incubated with 2 mM MG for 8 h.

### 2.4. Cell Viability

To determine cell viability, a 3-(4,5-dimethylthiazol-2-yl)-2,5-diphenyltetrazolium bromide (MTT) assay was conducted. Cells (1 × 10^5^ cells/well) were seeded in 96-well plates and incubated for 24 h at 37 °C. MTT solution (0.1 mg/mL) was added to the plates and maintained for 3 h. After that, the MTT solution was discarded, and DMSO was added to dissolve formazan crystals. Absorbance was measured at a wavelength of 540 nm using a multiplate reader (ELx808, BioTek, Winooski, VT, USA).

### 2.5. Glo-1 Enzyme Activity

Cells were washed twice with PBS and harvested in PBS on 1.5-mL tubes. Cell suspensions were homogenized in ice-cold phosphate-buffered saline using ultrasonication, and the homogenates were centrifuged at 13,000× *g* for 15 min at 4 °C. The supernatants were stored at −20 °C until further use. The reaction mixture comprised 2 mM GSH and 2 mM MG in 50 mM sodium phosphate buffer (pH 6.6). It was pre-incubated for 10 min at 37 °C to generate hemithioacetal adducts. After that, 20 μL of each sample and 180 μL of the reaction mix were added to 96 well UV plates (SPL Lifesciences, Gyeonggi, Korea). The following SLG production was estimated by measuring absorbance at 240 nm for 30 min and corrected based on protein contents.

### 2.6. Western Blotting

Cells were lysed using RIPA buffer, and the lysates were centrifuged at 15,000 rpm for 20 min at 4 °C, after which supernatants were collected. Total protein concentration was measured using the BCA protein assay kit from ThermoFisher Inc. (Carlsbad, CA, USA). Equal amounts of protein (20 μg per sample) were electrophoresed on 10% SDS-PAGE gel. After the proteins were transferred to Polyinylidene fluoride Immobilon membranes (Merck Millipore, Billerica, MA, USA), non-specific binding was blocked using 5% (*w/v*) skim milk prepared in Tris-buffered saline-Tween detergent (TBST) solution [50 mM tris-hydroxymethyl amino methane, pH 7.5 containing 1 mM MgCl_2_, 10 mM NaCl, 0.1 g/L NaN_3_, and 1 g/L Tween-20 (Sigma Chemical Co., MO, USA) for 45 min and probed overnight with primary antibodies (1:1000) at 4 °C. After washing the membranes with TBST for 30 min, horseradish peroxidase-conjugated secondary antibodies (1:4000) were used to probe the membrane for 1 h. Then, the membranes were washed again and the bands were visualized by enhanced chemiluminescence kit (Bio-Rad, Hercules, CA, USA) and ChemiDoc (Bio-Rad, Hercules, CA, USA), the expressions were quantified by Image J analysis software, version 1.51k (Bethesda, MD, USA).

### 2.7. ROS Production

Cells were seeded at a density of 1 × 10^5^ cells/well in 96 black well plates and incubated at 37 °C. After the pre-treatment of CA and NAC, cells were washed with PBS and incubated with 100 μM 2′,7′-dichlorodihydrofluorescein diacetate (DCF-DA) for 30 min. After removing DCF-DA, cells were incubated with phenol red-free RPMI-1640 medium containing 2.0 mM MG. Then, intracellular ROS production was measured based on the fluorescence intensity of excitation/emission at 485/535 nm in Hidex sense microplate reader (Hidex, Turku, Finland).

### 2.8. RNA Isolation

To isolate total RNA, cells were washed with PBS twice and homogenized using RNAiso PLUS (Takara, Seoul, Korea). Following that, cDNA was synthesized using the cDNA synthesis kit (Legene, CA, USA) according to the manufacturer’s instructions.

### 2.9. Quantitative Reverse Transcriptase-Polymerase Chain Reaction (qRT-PCR)

qRT-PCR was performed using 20 μL of reactions of primer, cDNA, and SYBR Green (Enzynomics, Dajeon, Korea) using iQ5 Thermal Cycler (Bio-Rad, Hercules, CA, USA). The cycle threshold (Ct) values were normalized with respect to the house-keeping gene (β-actin), and calculated using the 2^−*ΔΔCt*^ method. Primer sequences are shown in Table 1.

### 2.10. Mitochondrial Membrane Potential (MMP) Assay

MMP was measured using JC-1 dye. Cells were seeded at a density of 1 × 10^5^ cells/well in black 96-well plates for fluorimetry and glass-bottom dishes for live-cell imaging. Cells were washed with PBS once and stained with 4 μM JC-1 dissolved in medium for 20 min. JC-1 was removed by washing with PBS twice. Fluorescence intensity was measured using Hidex Sense microplate reader (Hidex, Turku, Finland) and confocal laser scanning microscopy LSM700 (Carl Zeiss, Oberkochen, Germany).

### 2.11. ATP Synthesis

White 96-well cell culture plates were used, and cells were seeded at a density of 5 × 10^5^ cells/well. To determine synthesized ATP contents, the ATPlite luminescence assay kit (Cat. No: 6016943) from Perkin Elmer (Billerica, MA, USA) was employed according to the manufacturer’s instructions.

### 2.12. Glucose-Stimulated Insulin Secretion (GSIS) Assay

INS-1 cells were cultured at a density of 1 × 10^5^ cells/well in 24-well plates. Cells were washed twice with Kreb’s ringer bicarbonate (KRB) buffer (137 mM NaCl, 4.7 mM KCl, 2.5 mM CaCl_2_, 1.2 mM MgSO_4_, 1.2 mM KH_2_PO_4_, 25 mM NaHCO_3_, and 1% BSA) (pH 7.4) and starved for 1 h with the same buffer. Then, cells were stimulated using a KRB buffer containing 2.8 mM and 22 mM of glucose for 1 h. The supernatant was collected from each well and was used to measure the insulin levels. The rat/mouse insulin ELISA kit (Cat. No: EZRMI-13K) from Merck-Millipore (St. Louis, MO, USA) was used according to the manufacturer’s instructions.

### 2.13. Statistical Analysis

The data are expressed as mean ± S.D. values of three independent experiments. Experiments were repeated three times (*n* = 3). Different letters mean significant differences at *p* < 0.05 according to ANOVA followed by the Tukey’s multiple range tests. Differences between means that share a letter are not statistically significant. Statistical analyses were performed using SAS version 9.4 (SAS Institute, Cary, NC, USA).

## 3. Results

### 3.1. CA Prevents MG-Induced Loss of INS-1 Cell Viability

**Figure 2:**
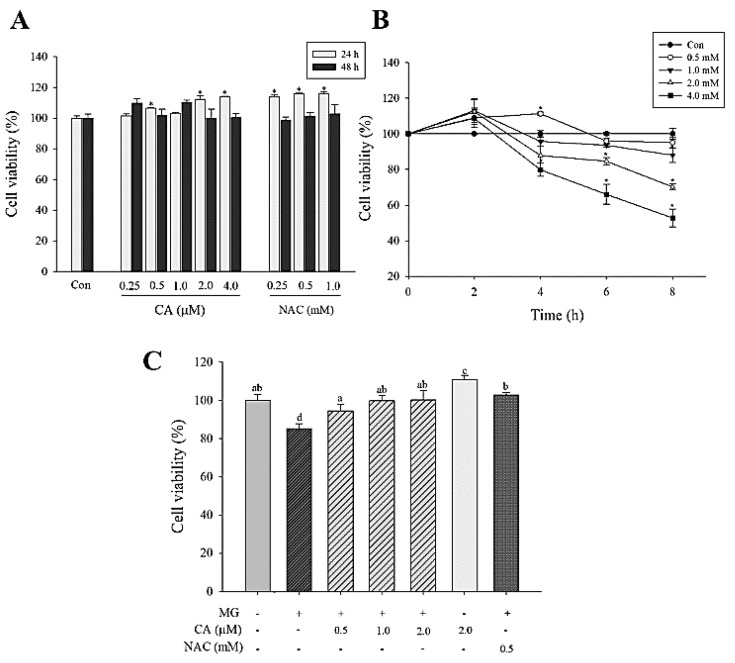
Viability of INS-1 cells treated with CA, N-acetyl-cysteine (NAC), and methylglyoxal (MG). (**A**) Cells treated with various concentrations of CA (0.25–4.0 μM) and NAC (0.25–1.0 mM) for 24 and 48 h. (**B**) Cells treated with various concentrations of MG (0.5–4.0 mM) for 2–8 h. (**C**) Effect of CA pre-treatment for 48 h on the viability of INS-1 cells. INS-1 cells were pre-treated with CA, NAC, and medium and then incubated with 2.0 mM MG for 8 h. The data are expressed as mean ± S.D. values of three independent experiments. Different letters indicate significant differences at *p* < 0.05 according to Tukey’s multiple range test. Differences between means that share a letter are not statistically significant.

CA (0.25–4.0 μM) and NAC (0.25–1.0 mM) treatments lasting for 24 and 48 h did not exhibit any cytotoxicity (Figure 2A). NAC has been used as a positive control. MG treatment (0.5–4.0 mM) for 4–8 h reduced cell viability in a time- and concentration-dependent manner. In the 2.0 mM MG-treated cells for 8 h, cell viability was significantly (*p* < 0.05) decreased to 70% (Figure 2B). We found that the pre-treatment with CA (0.5–2.0 μM) and NAC (0.5 mM) for 48 h significantly (*p* < 0.05) mitigated MG-associated loss of INS-1 cell viability (Figure 2C).

### 3.2. CA Recovers the MG-Induced Down-Regulated Glo-1 Protein Expression and Enzyme Activity

**Figure 3:**
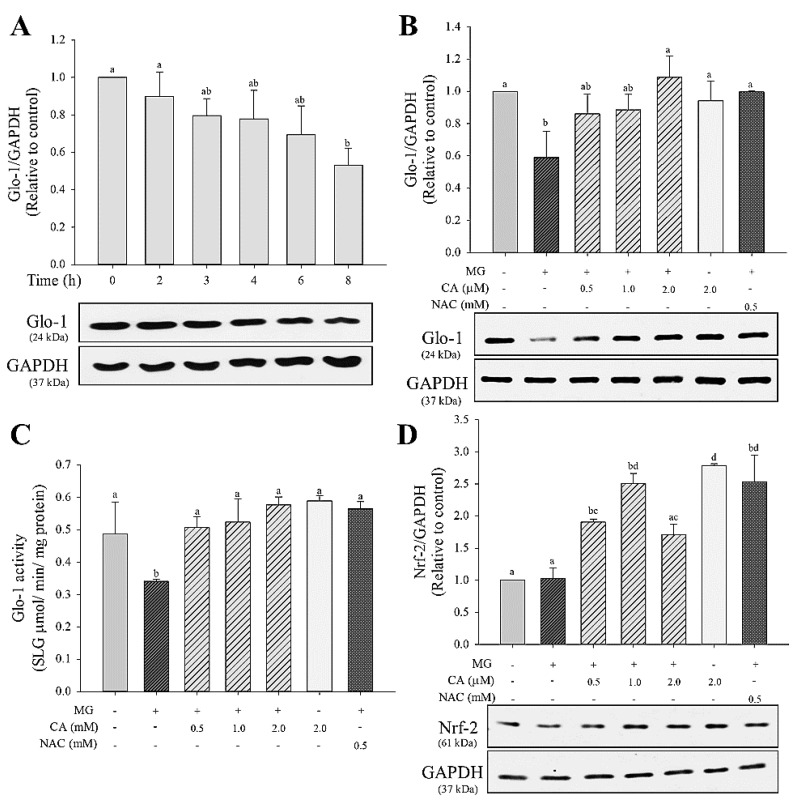
Effect of CA on decreasing glyoxalase-1 (Glo-1) protein expression and MG enzyme activity. Protein expression of Glo-1 in INS-1 cells treated with (**A**) 2.0 mM MG for 2–8 h and (**B**) pre-treated with CA, NAC, and medium for 48 h and then treated with 2.0 mM MG for 8 h. (**C**) The effect of CA on Glo-1 enzyme activity; (**D**) nuclear factor erythroid 2-related factor 2 (Nrf-2) protein expression in INS-1 cells treated with 2.0 mM MG for 8 h following pre-treatment with CA, NAC, and medium for 48 h. Glo-1 activity is represented as the production of S-d-lactolyglutathione per mg of protein in 1 min. The data are expressed as mean ± S.D. values of three independent experiments. Different letters indicate significant differences at *p* < 0.05 according to Tukey’s multiple range test. Differences between means that share a letter are not statistically significant.

The treatment of INS-cells with 2 mM MG (2–8 h) lowered the protein expression of Glo-1 in a time-dependent manner, with a significant (*p* < 0.05) decrease (about 50%) at 8 h (Figure 3A). Cells pre-treated with CA (0.5–2.0 μM) or NAC (0.5 mM) for 48 h restored the level of Glo-1 protein expression to that of untreated control cells (Figure 3B). Further, as shown in Figure 3C, Glo-1 enzyme activity significantly (*p* < 0.05) decreased to 0.3 mmol/min/mg protein in MG-only treated cells, whereas the CA- or NAC-pre-treated cells showed fully recovered Glo-1 activity (0.5–0.6 mmol/min/mg protein) compared with that of untreated control cells. The observed Nrf-2 protein expression is presented in Figure 3D. No significant (*p* < 0.05) difference in Nrf-2 expression was found in the MG-only treated group. However, under conditions of CA (0.5–2.0 μM) and NAC (0.5 mM) pre-treatments, Nrf-2 expression was upregulated to about 1.5 times that of the control group.

### 3.3. CA Prevents MG-Induced ROS Production

**Figure 4:**
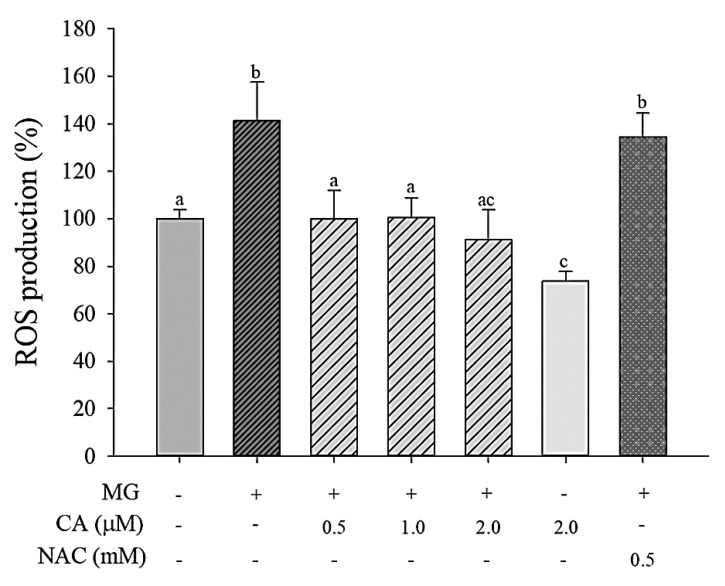
Effect of CA on ROS production in INS-1 cells incubated with 2.0 mM MG for 8 h following CA, NAC, and medium pre-treatment for 48 h. Values are presented as mean ± SD (*n* = 3), and different letters indicate significant differences at *p* < 0.05.

To evaluate the antioxidant abilities of CA, cellular ROS production was measured (Figure 4). When INS-1 cells were treated with 2 mM MG for 8 h, ROS production increased about 40% compared with that of the control cells. However, ROS formation was significantly (*p* < 0.05) decreased upon the pre-treatment of cells with CA (0.5–2.0 μM); NAC pre-treatment (0.5 mM) did not reduce MG-induced ROS production.

### 3.4. CA Regulates the MG-Induced mRNA Expression of Mitochondrial Dysfunction Markers

**Figure 5:**
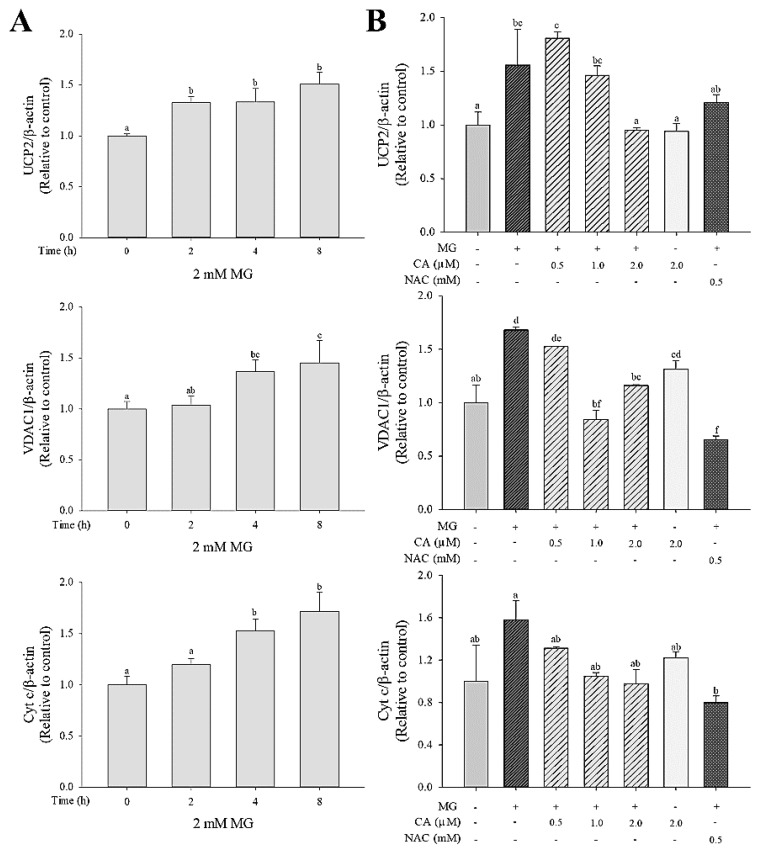
Effect of CA on mRNA expression of mitochondrial dysfunction related-components (UCP2, VDAC1, and cyt *c*) in INS-1 cells (**A**) treated with 2.0 mM MG for 2–8 h and (**B**) pre-treated with CA, NAC and medium for 48 h and then treated with 2.0 mM MG for 8 h. Values are presented as mean ± SD (*n* = 3), and different letters indicate significant differences at *p* < 0.05.

We investigated the effect of MG on the mRNA expression of mitochondrial proteins, including uncoupling protein 2 (UCP2), voltage-dependent anion-selective channel protein-1 (VDAC1), and cytochrome c (cyt *c*). Upregulation of UCP2, VDAC1, and cyt *c* mRNA was observed in cells treated with 2.0 mM MG for 2 to 8 h (Figure 5A). This MG-induced UCP2 and VDAC1 mRNA expression was significantly (*p* < 0.05) decreased upon pre-treatment with CA, but no significant (*p* < 0.05) difference in cyt *c* mRNA expression was observed among the untreated control cells, MG-only-treated cells, and CA-pre-treated cells (Figure 5B).

### 3.5. CA Upregulates MG-Induced MMP Loss

**Figure 6:**
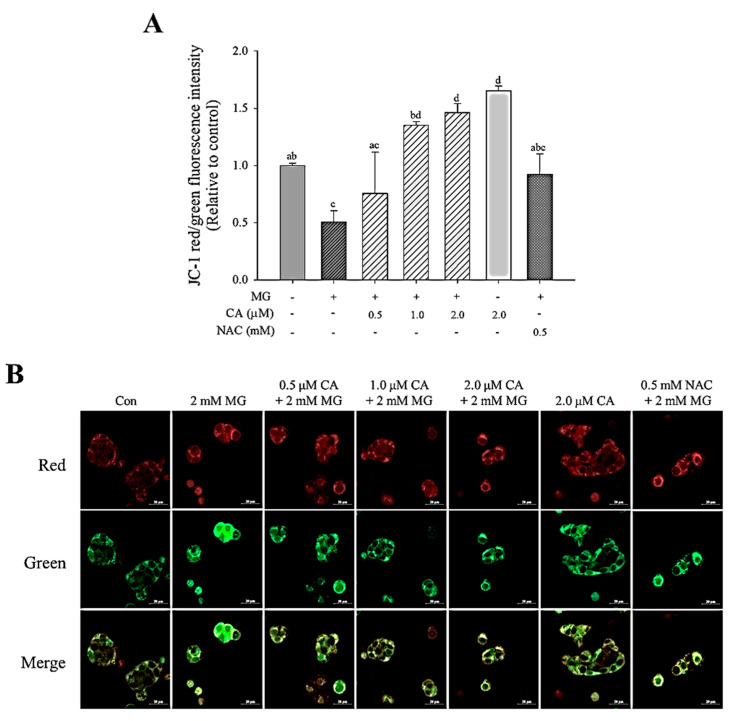
Effect of CA on MG-induced mitochondrial membrane potential (MMP) decrease determined using (**A**) a fluorometer and (**B**) confocal microscopy. INS-1 cells were treated with 2 mM MG for 8 h after pre-treatment with CA, NAC, and medium for 48 h. The fluorescence of J-aggregates (red) and J-monomers (green) was measured at excitation/emission wavelengths of 535/590 and 485/535 nm, respectively. Data are shown as the ratio of red to green. Values are presented as mean ± SD (*n* = 3), and different letters indicate significant differences at *p* < 0.05.

Mitochondrial membrane potential was measured to evaluate the protective effect of CA against MG-induced mitochondrial dysfunction. As shown in Figure 6A, treatment with 2.0 mM MG for 8 h yielded significantly (*p* < 0.05) lower red/green fluorescence intensity (less than 50%) than the untreated control. Additionally, significant (*p* < 0.05) increases in MMP were observed in cells pre-treated with 1.0 μM and 2.0 μM CA. In confocal images (Figure 6B), the MG-induced green fluorescence intensity was suppressed in CA-pre-treated cells.

### 3.6. CA Prevents MG-Induced Loss of ATP Synthesis

**Figure 7:**
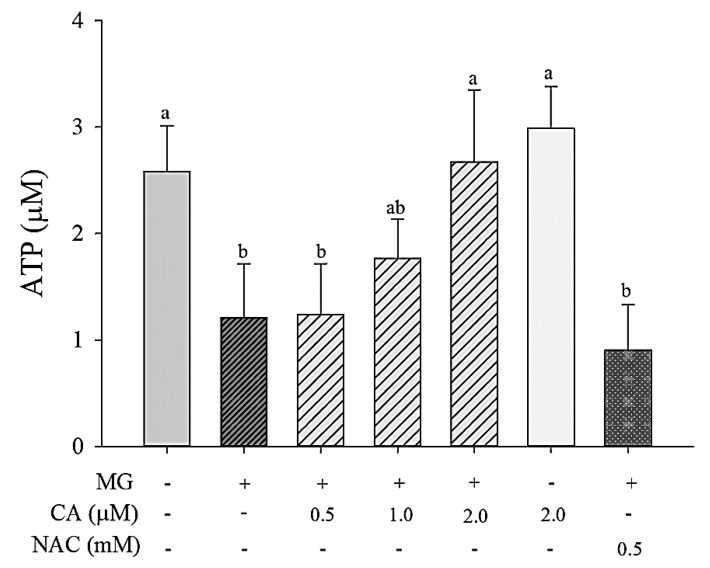
Effect of CA on MG-induced decrease of ATP synthesis. INS-1 cells were pre-treated with 2 mM MG for 8 h followed by CA, NAC and medium for 48 h. The amount of synthesized ATP was measured using a luminometer. Values are presented as mean ± SD (*n* = 3), and different letters indicate significant differences at *p* < 0.05.

The effect of CA pre-treatment prior to MG treatment on ATP synthesis was assessed. Cells treated with MG showed a significantly (*p* < 0.05) decreased ATP synthesis (1.0 μM ATP, which was 53% lower than that in control cells), whereas the amount of ATP was fully (*p* < 0.05) recovered upon pre-treating the cells with 2.0 μM CA (Figure 7). Pre-treatment with 0.5 mM NAC failed to prevent MG-induced synthesis reduction.

### 3.7. CA Prevents MG-Induced GSIS Decrease

**Figure 8:**
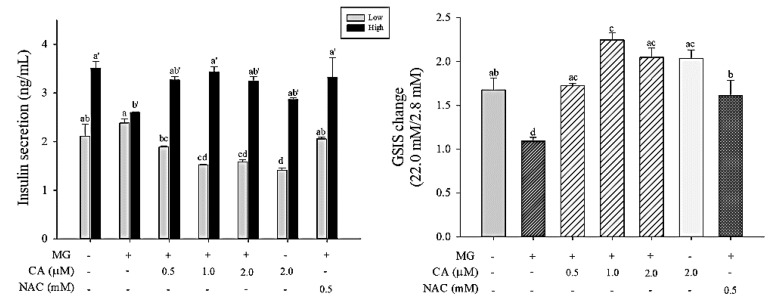
Effect of CA on MG-induced decrease of glucose-stimulated insulin secretion (GSIS) at low (2.8 mM) or high (22.0 mM) of glucose and the fold change of insulin secretion from 2.8 mM to 22.0 mM. INS-1 cells were pre-treated with 2 mM MG for 8 h after treatment with CA, NAC, and medium for 48 h. Absorbance was measured at a wavelength of 450 nm, corrected by 590 nm. Values are presented as mean ± SD (*n* = 2), and different letters indicate significant differences at *p* < 0.05.

Insulin secretion in response to a low dose of 2.8 mM glucose and a high dose of 22.0 mM glucose was measured in INS-1 cells (Figure 8). A slight increase in insulin secretion in INS-1 cells treated with 2 mM MG for 8 h was observed in response to both low and high glucose doses. However, when INS-1 cells were pre-treated with CA and NAC, GSIS increased up to the level of the control in response to a high dose of glucose (22.0 mM).

## 4. Discussion

The present study showed that MG decreased β-cell viability with increasing concentration and exposure time, which is consistent with the previous study [14]. Pancreatic β-cell (INS-1E) viability decreased in a concentration- (0.05–1.0 mM) and time- (0–24 h) dependent manner; viability was observed to decrease by 80% when exposed to 1.0 mM MG for 24 h [14]. Short-term treatment with high concentrations of MG has been reported to induce intracellular damage similar to that of hyperglycemia [15]. Therefore, via the glyoxalase pathway, the cellular detoxification of the reactive dicarbonyl metabolite, MG is essentially implemented [16,17]. In this pathway, Glo-1, a major MG detoxifying enzyme, catalyzes the first and the rate-limiting step in the removal of MG, and is crucial in protecting cells against oxidative stress [18,19]. A lack of Glo-1 elicits damage to mitochondria due to increased ROS production [20]. The linkage between a lack of Glo-1 and ROS production, inflammation, and apoptosis has also been causatively demonstrated [5,21,22,23]. Xue et al. [24] reported that transcriptional factor Nrf2 is involved in upregulating Glo-1 expression through a functional antioxidant-responsive element (ARE) in exon 1 of the gene. Activation of Nrf2 and the upregulation of its downstream targets heme oxygenase-1 and Glo1 is associated with loss of function of KRIT1 (CCM1) [25]. Liu et al. [26] reported that the treatment of 10–20 μM quercetin, dietary flavonoid widely distributed in fruits and vegetables, significantly increased protein, mRNA, and activity of Glo-1 in SH-SY5Y neuronal cells with a high glucose dose (70 mM); it increased Nrf2 nucleus translocation and elevated the protein and mRNA levels of γ-glutamylcysteinesynthetase (γ-GCS), a well-known Nrf2/ARE signaling target gene.

A few studies documented the protective effect of phytochemicals, including cyanidin, deoxyactin, and sciadopitysin against MG-induced oxidative stress in pancreatic β-cells [27,28,29]. The authors showed that those flavonoid treatment in the β-cells recovers Glo-1 activity inhibited by MG, although their modulation of Nrf2 on Glo-1 expression in the cells was not studied. In the present study, Glo-1 protein expression and enzyme activity were down-regulated by MG treatment, but this effect was reversed by pre-treatment with CA in the INS-1 β-cells. Glo-1 activity is highly correlated with insulin sensitivity [18], and hyperglycemia and insulin resistance were increased in Glo-1 knockout animals [30]. Previous studies reported that MG treatment decreases Glo-1 activity in a time-dependent manner in pancreatic β-cells, and that isoferulic acid treatment (50–100 μM) attenuates MG-induced cell death by increasing Glo-1 activity [31]. Similarly, deoxyactein (0.01 and 0.1 μM) restored MG-reduced Glo-1 activity and insulin secretion in RIN-m5F cells [30]. In line with these results, our study suggests that CA protects β-cells against MG-induced toxicity by increasing Glo-1 protein expression and enzyme activity. We also observed an increase in Nrf2 protein expression in the INS-1 cells pre-treated with CA, although a similar effect by another phytochemical, resveratrol has been described [32]. Thus, it is highly possible that the CA pre-treatment of INS-1 cells may increase Glo-1 protein expression and enzymatic activity via the Nrf2 signaling pathway. Similarly, the redox-dependent upregulation of Nrf2-mediated Glo-1 to protect human lung cells against gold nanoparticle-induced toxicity has been reported [33]. Human endothelial venous umbilical cells (HUVEC) treated with 10 μM CA prior to glyceraldehyde-induced AGEs treatment showed a substantial improvement in nuclear Nrf2 level through activation of extracellular signal-regulated kinase (ERK), and oral administration of 10 mg of CA/kg b.w. for 2 weeks and 5 d at the same time injected with AGEs showed improved Nrf2 expression [12]. It remains of interest to investigate whether CA protects against MG-induced mitochondrial dysfunction via the mediation of ERK-Nrf2 pathway and its downstream target Glo1 in pancreatic β-cells.

Our previous studies have demonstrated CA’s anti-oxidant ability and its effectiveness in preventing hepatic fibrosis, lung damage owing to urban particulate matter, and vascular dysfunction [11,12,13]. In further agreement with previous studies, we also observed that CA (0.5–2.0 μM) significantly (*p* < 0.05) prevented the formation of ROS in INS-1 β-cells. However, molar-level pre-treatment with 0.5 mM NAC, a compound well-known as a free radical scavenger [34], did not suppress MG-induced ROS production significantly (*p* < 0.05) (Figure S1). NAC treatment, was used as a positive control at a molar concentration 250–1000 fold that of the CA level. These findings suggest that CA acts as a potent antioxidant while increasing Glo-1 and protecting the pancreas from MG toxicity. To date, no pharmacokinetic have investigated the peak blood concentration of CA in animals. However, Lu et al. [35] reported that chebulinic acid and chebulagic acid, which are O-glycosidic ellagitannins of CA, reached peak concentrations of 0.64 μM and 1.4 μM, respectively, at 54 min following intraperitoneal injection into male Sparague-Dawley rats. Because it is plausible that both compounds can be hydrolyzed to yield chebulic acid via metabolism in the body, the CA concentrations of 0.5–2 μM considered in this study seem to be in the range of physiologically possible plasma concentrations.

Mitochondrial dysfunction is related to changes of MMP, ATP synthesis, and GSIS [36]. UCP2, an electron transport protein located in mitochondrial inner membranes, has been studied as a mitochondrial dysfunction marker in β-cells [27,28]. Up-regulation of UCP2 protein expression has been observed in the pancreatic islets of diabetic ob/ob mice. Also, raised ATP levels and insulin secretion have been reported in UCP2-deficient pancreatic islets of mice [27]. Inhibition of UCP2 by genipin (50 μM) attenuates β-cell dysfunction by increasing MMP, ATP, and GSIS in pancreatic islets damaged by high glucose levels [37]. VDAC1, a protein located in mitochondrial outer membranes, contributes to MMP loss and the release of cyt *c*, a mitochondrial pro-apoptotic protein [38,39]. Thus, VDAC1 is considered a major target for preventing β-cell dysfunction and apoptosis. VDAC1 expression has been observed in INS-1E pancreatic β-cells under glucotoxic conditions [40]. Another study showed that the VDAC1-knockout pancreatic islets of db/db mice maintained insulin secretion and β-cell function [41]. Cyt *c*, a mitochondrial respiratory chain component, is reportedly secreted in high levels due to MG-induced ROS in INS-1 pancreatic β-cells [42]. Gelsolin (400 nM) attenuated mitochondrial dysfunction by inhibiting the release of cyt *c* in Jurkat cells [43]. In agreement with previous studies, we observed that 2 mM MG increased the mRNA expression of UCP2, VDAC1, and cyt *c* and that these increases were suppressed by CA pre-treatment (0.5, 1.0, and 2.0 μM) for 48 h; however, no significant differences were observed regarding cyt *c* levels. Although VDAC1 and cyt *c* are considered pro-apoptotic proteins [38], we have not yet studied how CA acts on the apoptotic pathway. Thus, further studies on this mechanism are needed.

Previous studies have found that MG treatment lowers MMP in a concentration-dependent manner, and reduced MMP lowers intracellular ATP levels in neuronal cells (SH-SY5Y) [4]. In pancreatic β-cells, MG reduces insulin secretion by decreasing MMP and ATP synthesis [14]. Similarly, we observed a loss of MMP after MG treatment and an associated reduction in ATP synthesis; however, these phenomena were alleviated by CA pre-treatment. In addition, our GSIS data showed that MG treatment reduced the insulin sensitivity of INS-1 cells stimulated with high glucose levels, but GSIS was alleviated by CA pre-treatment. MG treatment reduces insulin secretion in cells stimulated with high glucose (11.0 and 22.0 mM), and isoferulic-acid treatment attenuates MG-induced impairment of GSIS [31]. Although the amount of insulin secretion decreased with CA pre-treatment under normal glucose levels, CA did not affect GSIS. This could be attributable to slow insulin secretion. The mechanism of CA-induced changes in GSIS may require further study. However, our results suggest that CA can prevent MG-induced mitochondrial dysfunction and thereby protect β-cells against MG-induced damage by supporting β-cell sensitivity to insulin.

## 5. Conclusions

In this study, we identified the preventive effects of CA against MG-induced mitochondrial dysfunction in INS-1 pancreatic β-cells. CA prevented the loss of INS-1 cell viability and increased Nrf-2 protein expression, Glo-1 protein expression, and enzyme activity that was diminished by MG. Although more remains to be learned about causal link between oxidative stress and mitochondrial dysfunction in this study, increased Glo-1 lowered ROS production levels, thus possibly preserving mitochondrial function via the downregulation of UCP2, VDAC1, and cyt *c* and increased MMP and ATP synthesis. Ultimately, CA prevented the reduction of GSIS. These results suggest that CA could be employed as a therapeutic agent to prevent oxidative stress-induced β-cell dysfunction.

## Figures and Tables

**Table 1:** Sequences of qRT-PCR primers.

Gene	Accession Number	Primer Sequence (5′-3′)	
		Forward	Reverse
UCP2	NM_019354.3	GCA CTG TCG AAG CCT ACA AGA C	TGG CAT TTC GGG CAA CAT
VDAC1	NM_031353.1	GAC AAC ACC CTG GGC ACT G	CAC AGC CCA GGT TGA TAT G
cyt *c*	NM_012839.2	TGC CCA GTG CCA CAC TGT	CTG TCT TCC GCC CGA ACA
β-actin	NM_031144.3	TCA GGA GGA GCA ATG ATC TTG A	GAC AGG ATG CAG AAG GAG ATC AC

qRT-PCR quantitative reverse transcriptase-polymerase chain reaction, UCP2 uncoupling protein 2, VDAC1 voltage-dependent anion-selective channel-1, cyt *c*, cytochrome c.

## Supplementary Materials

The following are available online at https://www.mdpi.com/2076-3921/9/9/771/s1, Figure S1: Effect of NAC pretreatment for 48 h on the MG-induced ROS production in INS-1 cells.

## Abbreviations

AGEs	advanced glycation end products
ARE	antioxidant-responsive element
CA	chebulic acid
Ct	cycle threshold
cyt c	cytochrome c
DCF-DA	2′,7′-dichlorodihydrofluorescein diacetate
ERK	extracellular signal-regulated kinase
GAPDH	glyceraldehyde-3-phosphate dehydrogenase
γ-GCS	γ-glutamylcysteine synthetase
Glo-1	glyoxalase-1
GSH	glutathione
GSIS	glucose-stimulated insulin secretion
HEPES	4-(2-hydroxyethyl)-1-piperazineethane sulfonic acid
HUVEC	Human endothelial venous umbilical cells
MG	methyl glyoxal
MMP	mitochondrial membrane potential
MTT	3-(4,5-dimethylthiazol-2-yl)-2,5-diphenyltetrazolium bromide
NAC	N-acetyl-cysteine
Nrf-2	nuclear factor erythroid 2-related factor 2
qRT-PCR	quantitative reverse transcriptase-polymerase chain reaction
ROS	reactive oxygen species
SLG	S-D-lactoylglutathione
SOD	superoxide dismutase
TBST	tris-buffered saline-Tween detergent
UCP2	uncoupling protein 2
VDAC1	voltage-dependent anion-selective channel-1
