# Supplementary material for: Chebulic Acid Prevents Methylglyoxal-Induced Mitochondrial Dysfunction in INS-1 Pancreatic β-Cells
Source: Antioxidants (Basel). 2020 Aug 20;9(9):771. doi: 10.3390/antiox9090771 (PMC7554990; doi:10.3390/antiox9090771)

Supplementary material

Figure S1. Effect of NAC pretreatment for 48 h on the MG-induced ROS production in INS-1cells incubated with 2.0 mM MG for 8 h Values are presented as mean ± SD (n=3) and different letters mean significant differences at p <0.05.

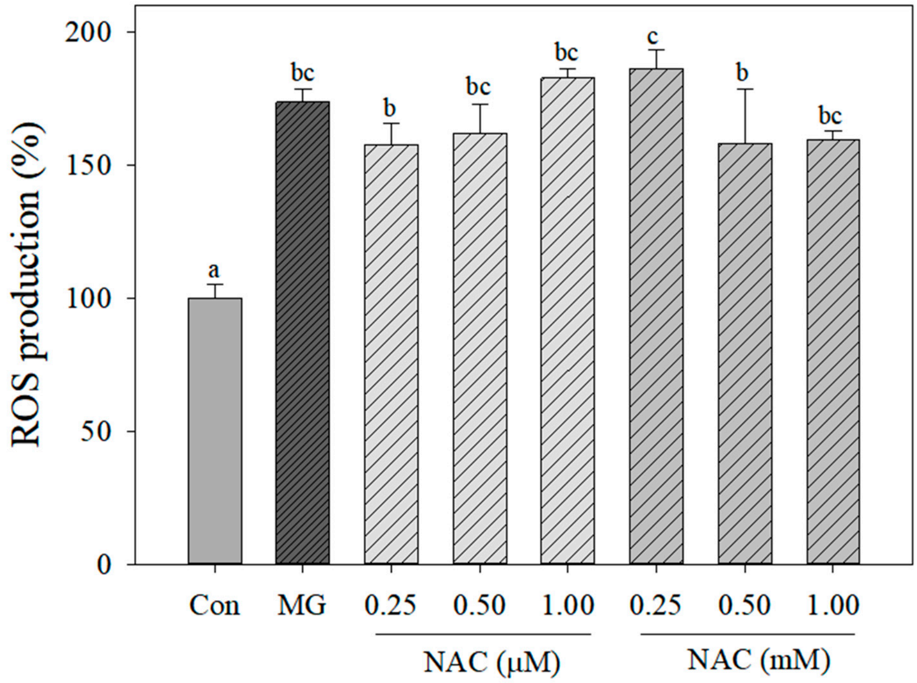

Supplement: Supplementary file 1 [file antioxidants-09-00771-s001.pdf]
